# Arterial dP/dt_max_ Response to Fluids and Norepinephrine During Intraoperative Hypotension: A Prospective Physiological Study

**DOI:** 10.3390/jcm15166471

**Published:** 2026-08-21

**Authors:** Andrea Russo, Manuel Ignacio Monge García, Antonio Maria Dell’Anna, Tiziano Torce’, Salvatore Silvio Melcore, Giuseppe Romano, Francesco Scialanga, Tiziana Iacobucci, Flaminio Sessa, Tiziana Bove, Massimo Antonelli, Paola Aceto

**Affiliations:** 1 Department of Emergency, Anesthesiologic and Reanimation Sciences, Fondazione Policlinico Universitario Agostino Gemelli IRCCS, 00168 Rome, Italy; 2 Unidad de Cuidados Intensivos, Hospital Universitario de Puerto Real, 11510 Puerto Real, Spain; 3 Department of Basic Biotechnological Science, Intensive and Peri-Operative Clinics, Università Cattolica del Sacro Cuore, 00168 Rome, Italy

**Keywords:** hemodynamic monitoring, intraoperative hypotension, fluid administration, preload dependence, major abdominal surgery

## Abstract

**Background:** Intraoperative hypotension during major abdominal surgery is common and can result from changes in preload, afterload, or myocardial contractility. Recognising the underlying cause is essential for targeted haemodynamic management. Arterial dP/dt_max_ has been proposed as a surrogate marker for ventricular systolic function, but its behaviour during fluid and vasopressor administration under general anaesthesia remains not fully understood. This study evaluated changes in radial arterial dP/dt_max_ following protocol-guided fluid administration and norepinephrine boluses during intraoperative hypotension. **Methods:** This prospective observational study involved 88 adult patients undergoing elective major abdominal surgery with continuous radial arterial waveform monitoring. Hypotensive episodes were defined as a mean arterial pressure <65 mmHg persisting for at least 60 s and were managed according to a predefined algorithm based on stroke volume variation and dynamic arterial elastance. The primary outcome was the change in arterial dP/dt_max_ from baseline to 5 min after fluid administration. Linear mixed-effects models were used to account for repeated intervention episodes within patients. **Results:** A total of 502 protocol-guided intervention episodes contributed by 76 patients were included in the primary analysis: 228 fluid-treated episodes and 274 norepinephrine-treated episodes. Estimated mean arterial dP/dt_max_ increased from 517 mmHg·s^−1^ (95% CI 477–557) at baseline to 631 mmHg·s^−1^ (95% CI 591–671) at 5 min during fluid-treated episodes and from 575 mmHg·s^−1^ (95% CI 536–615) to 647 mmHg·s^−1^ (95% CI 608–686) during norepinephrine-treated episodes (both *p* < 0.001). A significant intervention-condition-by-time interaction was observed (F = 5.55, *p* = 0.019), indicating that temporal evolution of arterial dP/dt_max_ differed between the two algorithm-defined haemodynamic conditions. **Conclusions:** Radial arterial dP/dt_max_ increased after both fluid administration and norepinephrine, confirming its sensitivity to haemodynamic loading conditions. It should therefore be interpreted as an integrated haemodynamic variable influenced by ventricular performance, preload, and arterial properties rather than as a direct measure of intrinsic myocardial contractility.

## 1. Introduction

Intraoperative hypotension (IOH) is a major determinant of postoperative organ dysfunction and adverse outcomes [1,2,3,4,5,6]. Although mean arterial pressure (MAP) is the most widely used target for intraoperative haemodynamic optimisation, it represents only a static measure of arterial pressure. MAP results from the interaction between ventricular function and the arterial system, but it does not provide insight into the underlying physiological mechanisms or dynamic determinants of this ventricular–arterial interaction.

Among arterial waveform-derived parameters, the maximum rate of arterial pressure rise, expressed as dP/dt_max_, has been proposed as a surrogate for left ventricular contractility [7,8,9]. Experimental and clinical data suggest that arterial dP/dt_max_ tracks changes in systolic function; however, its value is also influenced by preload and afterload, and its physiological determinants during anaesthesia remain incompletely understood. While maximum left ventricular dP/dt_max_ is traditionally measured centrally via invasive ventriculography and reflects intrinsic myocardial contractility under stable preload conditions, peripheral arterial dP/dt_max_ (such as that derived from the radial artery) should be considered a surrogate rather than a direct measure of ventricular performance. As the arterial pressure waveform propagates from the central aorta to the periphery, it undergoes progressive amplification and is shaped by wave reflections, vascular compliance, and arterial impedance. Consequently, peripheral arterial dP/dt_max_ reflects not only ventricular inotropy but also the complex interaction between stroke volume, arterial tone, and vascular properties. Compared with LV dP/dt_max_, it is therefore more sensitive to changes in preload and afterload. This distinction should be carefully considered when interpreting this parameter or when using it to guide haemodynamic management. Intraoperative management of IOH typically relies on two main therapeutic strategies: volume expansion to correct preload deficits and vasopressor administration to restore vascular tone. While fluid loading primarily increases SV and cardiac output (CO), vasopressors enhance MAP through increased arterial tone and may also increase SV by augmenting preload via the Frank–Starling mechanism. As arterial dP/dt_max_ is modulated by both contractility and loading conditions, its response to these interventions may differ. Characterising these responses could help clinicians interpret dP/dt_max_ changes within an individualised haemodynamic assessment.

Previous studies have explored the behaviour of arterial dP/dt_max_ in different clinical settings, mostly during preload-modifying manoeuvres or pharmacologic inotropy, but comparative data describing its response to fluids and vasopressors under anaesthesia are lacking [10,11]. Moreover, the extent to which changes in dP/dt_max_ reflect true contractile adaptation rather than load dependence has not yet been fully established in the perioperative setting.

This prospective physiological study primarily aimed to assess the effect of fluid administration on the radial arterial dP/dt_max_ and, secondarily, its response to vasopressor administration in anaesthetised patients undergoing major abdominal surgery, within a predefined haemodynamic optimisation algorithm based on continuous arterial waveform analysis.

## 2. Materials and Methods

This was an investigator-initiated, prospective physiological study conducted in the general surgery operating rooms of A. Gemelli University Hospital Foundation IRCCS, Rome, Italy. Adult patients undergoing general anaesthesia for elective major abdominal surgery (pancreaticoduodenectomy, Whipple procedure) were consecutively enrolled between February 2024 and March 2025. Ethical approval for this study (ID 6166) was provided by the Ethical Committee of CET Lazio Area 3 (Chairperson Prof. Andrea Bacigalupo) on 9 November 2023. The study has been registered at ClinicalTrials.gov (ID: NCT06164405). Written informed consent was obtained from all participants before surgery.

### 2.1. Patient Selection

Inclusion criteria were age ≥ 18 years, elective open abdominal surgery with an expected duration > 3 h, and planned use of continuous arterial pressure monitoring.

Exclusion criteria were body mass index (BMI) > 30 kg/m^2^; atrial fibrillation; congestive heart failure with left ventricular ejection < 35% and/or New York Heart Association (NYHA) class > III; severe valvular heart disease; emergency surgery; and preoperative shock or any acute conditions requiring admission to the intensive care unit.

### 2.2. Primary Endpoint

The primary endpoint was the variation in arterial dP/dt_max_ values after fluid challenge (after 5 min).

### 2.3. Secondary Endpoints

Secondary endpoints included the following:

1. Variation in arterial dP/dt_max_ 10 min after fluid administration.
2. Variation in arterial dP/dt_max_ 5 and 10 min after norepinephrine (NE) boluses.
3. Variation in arterial dP/dt_max_ after fluid administration stratified by stroke volume variation (SVV) quartiles at 5 and 10 min.
4. Effects of fluid and NE administration on stroke volume (SV), stroke volume variation (SVV), total arterial elastance (Eatot), and dynamic arterial elastance (Ea_dyn_).
5. Total intraoperative amounts of administered fluids and vasoactive drugs.

### 2.4. Anaesthesia Management

Anaesthesia was performed in accordance with the institutional protocol. General anaesthesia was induced with intravenous fentanyl (1.5–2 mcg/kg), propofol (1.5–2 mg/kg) and rocuronium (0.6 mg/kg). After tracheal intubation, volume control ventilation was initiated with a tidal volume of 8 mL/kg ideal body weight.

Maintenance of anaesthesia was achieved with sevoflurane (approximately 1 minimum alveolar concentration) in a mixture of 35% oxygen and 75% air at a fresh gas flow of 2 L/min, together with a continuous remifentanil infusion (0.05–0.2 mcg/kg/min). Sevoflurane concentration was titrated to maintain the Bispectral Index (BIS) between 40 and 60. Additional rocuronium boluses were given to maintain deep neuromuscular block (post-tetanic count ≤ 2). Respiratory rate was adjusted to maintain normocapnia (PaCO_2_ 35–45 mmHg).

### 2.5. Data Collection

At enrolment, the following variables were recorded: demographic data (age, sex, weight, height, and BMI), type of surgery, American Society of Anesthesiologists (ASA) physical status, baseline laboratory values (haemoglobin, serum creatinine, and albumin), electrocardiographic rhythm, and baseline haemodynamic parameters (heart rate and non-invasive arterial pressure). Perioperative haemodynamic and treatment data were prospectively collected and entered into a dedicated database.

### 2.6. Haemodynamic Measurement

Continuous haemodynamic monitoring was performed using the HemoSphere platform (Edwards Lifesciences, Irvine, CA, USA) connected to a radial arterial catheter via the Acumen IQ sensor (Edwards Lifesciences, Irvine, CA, USA), which provided pulse-contour-derived variables including SV, SVV, CO, Ea_dyn_, and arterial dP/dt_max_. The raw arterial pressure waveform was acquired by the HemoSphere platform at a sampling rate of 100 Hz, and all parameters were subsequently computed as 20 s averages spanning several respiratory cycles. Within this framework, dynamic arterial elastance (Ea_dyn_) was automatically calculated by the platform as the ratio between pulse pressure variation and stroke volume variation (PPV/SVV).

The invasive arterial pressure signal was routinely assessed by visual inspection and a square-wave (fast-flush) test by the attending anaesthesiologist. When underdamping or overdamping was detected, corrective measures were undertaken to optimise waveform quality. Only recordings with adequate waveform morphology and dynamic response characteristics were included in the analysis; non-interpretable waveforms were excluded.

### 2.7. Haemodynamic Algorithm and Interventions

Throughout the intraoperative period, any MAP value <65 mmHg generated an alert on the monitor. A hypotensive episode was formally defined as MAP <65 mmHg for at least 60 s. The episode was considered resolved once MAP returned to ≥65 mmHg following the protocol-defined haemodynamic intervention. No predefined minimum interval between consecutive hypotensive episodes was imposed. Any subsequent decrease in MAP below 65 mmHg lasting at least 60 s was considered a new hypotensive episode. Such episodes triggered algorithm-guided interventions based on SVV and E_dyn_ [12].

SVV ≥ 13% and Ea_dyn_ ≥ 0.9: a single 250-mL intravenous bolus of Ringer’s lactate was administered over 5 min according to the predefined haemodynamic algorithm. Haemodynamic variables were recorded 5 and 10 min after the start of fluid administration. When further fluid administration was subsequently indicated by the algorithm, each additional bolus was considered a separate intervention episode.

SVV ≥ 13% and Ea_dyn_ < 0.9: a 10-μg NE bolus was administered.

SVV < 13% with dP/dt_max_ <400 mmHg·s^−1^ (irrespective of Ea_dyn_): a 5 mg ephedrine bolus was given.

After the protocol-defined haemodynamic intervention, no additional haemodynamic treatment was administered during the subsequent 10-min observation period.

### 2.8. Follow-Up

All patients were followed up until hospital discharge.

### 2.9. Data Acquisition and Derived Analysis

Treatment allocation was based on the instantaneous haemodynamic values displayed by the monitor at the time the clinical decision was made. However, according to the study protocol, a hypotensive episode was formally defined as a mean arterial pressure < 65 mmHg persisting for at least 60 s. Because haemodynamic variables were automatically recorded every 20 s, the baseline value reported for each intervention episode was calculated as the arithmetic mean of the three consecutive measurements that defined the episode, rather than the single measurement that triggered treatment allocation. Consequently, the averaged baseline values of Ea_dyn_ and arterial dP/dt_max_ could differ from the instantaneous values used for treatment allocation and, in some cases, cross the predefined decision thresholds. Haemodynamic variables (MAP, SVV, Ea_dyn_, Eatot, and arterial dP/dt_max_) were subsequently recorded 5 and 10 min after the intervention. Total arterial elastance (Eatot) was calculated as 0.9 × SAP/SV [13,14].

### 2.10. Statistical Analysis

Sample size estimation was performed prospectively during study planning using the best available evidence at that time. Because no prior data were available specifically for the within-episode change in arterial dP/dt_max_ at 5 min after fluid administration, the effect reported by Vaquer et al. [15], based on the difference between fluid responders and non-responders, was used as an approximate basis for the calculation. The reported difference of 574 mmHg·s^−1^ with a standard deviation of 1279 mmHg·s^−1^ corresponded to a standardised effect size of 0.45. An a priori power analysis performed with G*Power version 3.1 (Heinrich Heine University, Düsseldorf, Germany), assuming a one-sided α of 0.05 and 90% power, indicated that 44 paired observations would be required. Based on the expected incidence of protocol-guided fluid administration, enrolment of 88 patients was considered sufficient to provide the anticipated number of evaluable fluid-treated episodes for the primary analysis.

Normality of data distribution was assessed using the Kolmogorov–Smirnov test. Continuous variables are expressed as mean ± standard deviation (SD) or median [interquartile range], as appropriate. Categorical data are presented as absolute numbers and percentages.

Because multiple hypotensive episodes could occur within the same patient, resulting in correlated observations, the primary inferential analyses were performed using linear mixed-effects models. Patient identity was included as a random intercept to account for within-patient correlation. For the primary analysis, fixed effects included intervention condition (fluid-treated versus norepinephrine-treated episodes), time (baseline and 5 min), and their interaction. In fluid-treated episodes, the SVV-stratified mixed-effects model included baseline SVV quartile, time (baseline and 5 min), and their interaction as fixed effects, with patient identity retained as a random intercept. Although observed changes at 10 min were retained for descriptive presentation, formal inferential analysis was restricted to the baseline-to-5-minute interval because temporally aligned observations within ±60 s of the prespecified 10 min assessment were not available for all intervention episodes. Parameters were estimated using maximum likelihood, and fixed effects were evaluated using Type III tests. The suitability of a model including a patient-specific random slope for time was assessed using likelihood ratio testing and by comparing the Akaike and Bayesian information criteria. Because no meaningful improvement in model fit was observed, the random-intercept model was retained for all subsequent analyses.

Conventional paired and between-group tests were used only for descriptive or secondary analyses of individual haemodynamic variables. All primary inferential conclusions regarding arterial dP/dt_max_ were based on linear mixed-effects models that accounted for repeated intervention episodes within patients.

Descriptive and conventional analyses were performed using IBM SPSS Statistics version 26 (IBM Corp., Armonk, NY, USA). Linear mixed-effects models and estimated marginal means were obtained using R version 4.5.2 (31 October 2025, ucrt), with the lme4 (version1.1-38), lmerTest (version 3.2-0) and emmeans (version 2.0.1) packages. Fixed effects were evaluated using Type III tests with Satterthwaite’s approximation for denominator degrees of freedom. Estimated marginal means, 95% confidence intervals, and pairwise contrasts were calculated using the Kenward–Roger method. Pairwise comparisons were adjusted using the Holm method where applicable. All tests were two-sided, and *p* values < 0.05 were considered statistically significant.

## 3. Results

**Figure 1:**
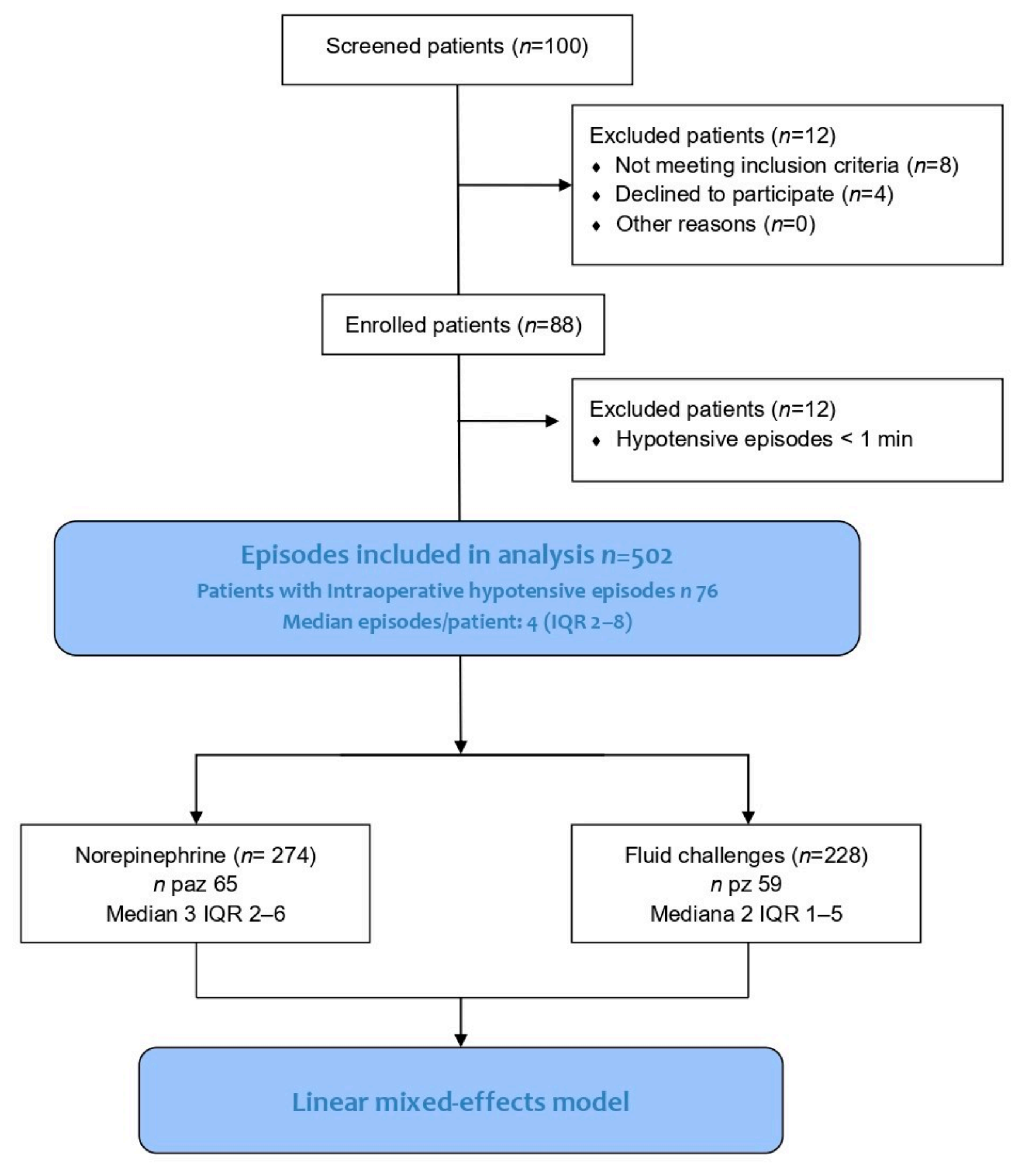
Flow diagram showing patient screening, eligibility assessment, enrolment, and protocol-guided intervention episodes. The diagram also reports the number of patients contributing to each intervention category and the distribution of repeated intervention episodes per patient. Individual patients could contribute multiple intervention episodes and could receive both fluid administration and norepinephrine during different hypotensive episodes. Only fluid- and norepinephrine-treated intervention episodes were included in the primary mixed-effects analysis. In addition, 29 patients experienced 59 episodes that met the predefined criteria for ephedrine administration; these episodes were not included in the primary mixed-effects analysis. The median number of episodes per patient was 1 (IQR 1–2; range 1–9).

Patient screening, eligibility assessment, and study enrolment are summarised in Figure 1. Patient-level variables were analysed in all enrolled patients (*n* = 88). Haemodynamic and physiological analyses were performed at the episode level and included only protocol-defined hypotensive episodes requiring algorithm-guided treatment with either fluid administration or norepinephrine. Of the 88 enrolled patients, 76 experienced at least one such episode, yielding a total of 502 intervention episodes (228 fluid-treated and 274 norepinephrine-treated) that were included in the primary mixed-effects analysis. Among these 76 patients, 29 also experienced 59 episodes meeting the predefined criteria for ephedrine administration. These episodes were summarised descriptively and were not included in the primary mixed-effects analysis [Figure S1].

Baseline demographic and intraoperative characteristics are summarised in Table 1. The median age was 70 years [63–75], and the median BMI was 24 kg/m^2^ [22–27]. Most patients were classified as ASA III (44.3%). The median duration of surgery was 327 min [271–418], with a median blood loss of 300 mL [200–500] and a urine output of 600 mL [378–988]. Intraoperative fluid administration consisted of 3500 mL [2500–4375] of crystalloids, and 42 patients (47.7%) additionally received 500 mL [250–750] of colloids. Blood transfusions were required in 17 patients (19.3%). Vasopressor therapy was frequent: NE was administered in 65 patients (73.9%) at a cumulative dose of 1000 µg [40–2400], and ephedrine was given in 29 patients (32.9%) at a cumulative dose of 10 mg [10–25]. Twenty-two patients (25%) required postoperative ICU admission, whereas 66 (75%) were transferred directly to the Post Anaesthesia Care Unit (PACU). The median hospital length of stay was 9 days [8–11]. Haemodynamic and metabolic parameters before and after surgery: cardiac output increased from 4.4 ± 1.5 L/min to 4.96 ± 1.4 L/min (*p* = 0.005) and arterial dP/dt_max_ increased from 678 ± 251 mmHg·s^−1^ to 746 ± 274 mmHg·s^−1^ (*p* = 0.01); lactate concentration decreased from 1.23 ± 0.86 mmol/L to 1.01 ± 0.40 mmol/L (*p* = 0.0006), while oxygen delivery (DO_2_) increased significantly from 631 ± 226 mL/min/m^2^ to 734 ± 264 mL/min/m^2^ (*p* = 0.001). No significant changes were observed in SV, SVV, or Ea_dyn_.

### 3.1. Changes in Arterial dP/dt_max_

The primary analysis was performed using a linear mixed-effects model accounting for repeated intervention episodes within individual patients. Significant effects of time (F = 105.6, *p* < 0.001), intervention condition (F = 10.8, *p* = 0.001), and the intervention-by-time interaction (F = 5.55, *p* = 0.019) were observed, indicating that the magnitude of the increase in arterial dP/dt_max_ from baseline to 5 min differed between fluid-treated and norepinephrine-treated episodes. A more complex model including a patient-specific random slope for time was also evaluated but did not provide a significantly better fit than the simpler random-intercept model (likelihood-ratio χ^2^ = 4.73, df = 2, *p* = 0.094; AIC 12,957.0 vs. 12,957.7; BIC 12,996.3 vs. 12,987.2). Therefore, the random-intercept model was retained for all subsequent analyses.

Estimated marginal means showed that arterial dP/dt_max_ increased from 517 mmHg·s^−1^ (95% CI 477–557) at baseline to 631 mmHg·s^−1^ (95% CI 591–671) at 5 min during fluid-treated episodes, corresponding to an estimated mean increase of 114 mmHg·s^−1^ (*p* < 0.001). During norepinephrine-treated episodes, arterial dP/dt_max_ increased from 575 mmHg·s^−1^ (95% CI 536–615) to 647 mmHg·s^−1^ (95% CI 608–686), corresponding to an estimated mean increase of 71.5 mmHg·s^−1^ (*p* < 0.001).

At baseline, estimated arterial dP/dt_max_ was 58.3 mmHg·s^−1^ higher during norepinephrine-treated than fluid-treated episodes (*p* < 0.001). At 5 min, the estimated difference was 15.7 mmHg·s^−1^ and was no longer statistically significant (*p* = 0.277).

**Figure 2:**
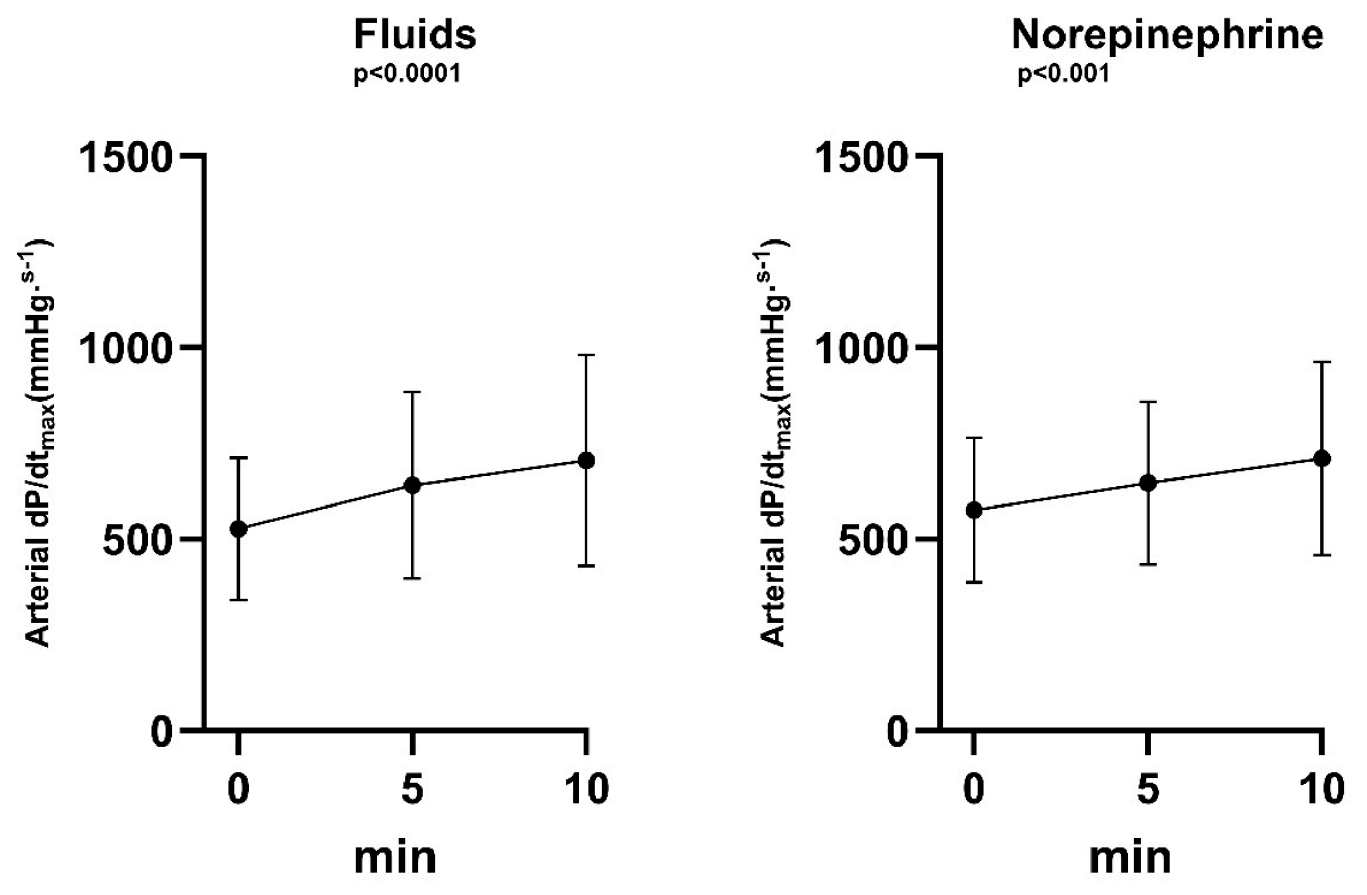
Time course of dP/dt_max_ following fluid or norepinephrine administration. Data are presented as mean ± SD at baseline and 5 and 10 min after the intervention.

The observed temporal evolution of arterial dP/dt_max_ is shown in Figure 2. In fluid-treated episodes, the observed mean value increased from 527 ± 186 mmHg·s^−1^ at baseline to 640 ± 244 mmHg·s^−1^ at 5 min and 705 ± 276 mmHg·s^−1^ at 10 min. In norepinephrine-treated episodes, the corresponding values were 575 ± 189, 647 ± 213, and 711 ± 253 mmHg·s^−1^, respectively. The 10 min findings are presented descriptively because the prespecified primary mixed-effects analysis evaluated the change at 5 min.

**Figure 3:**
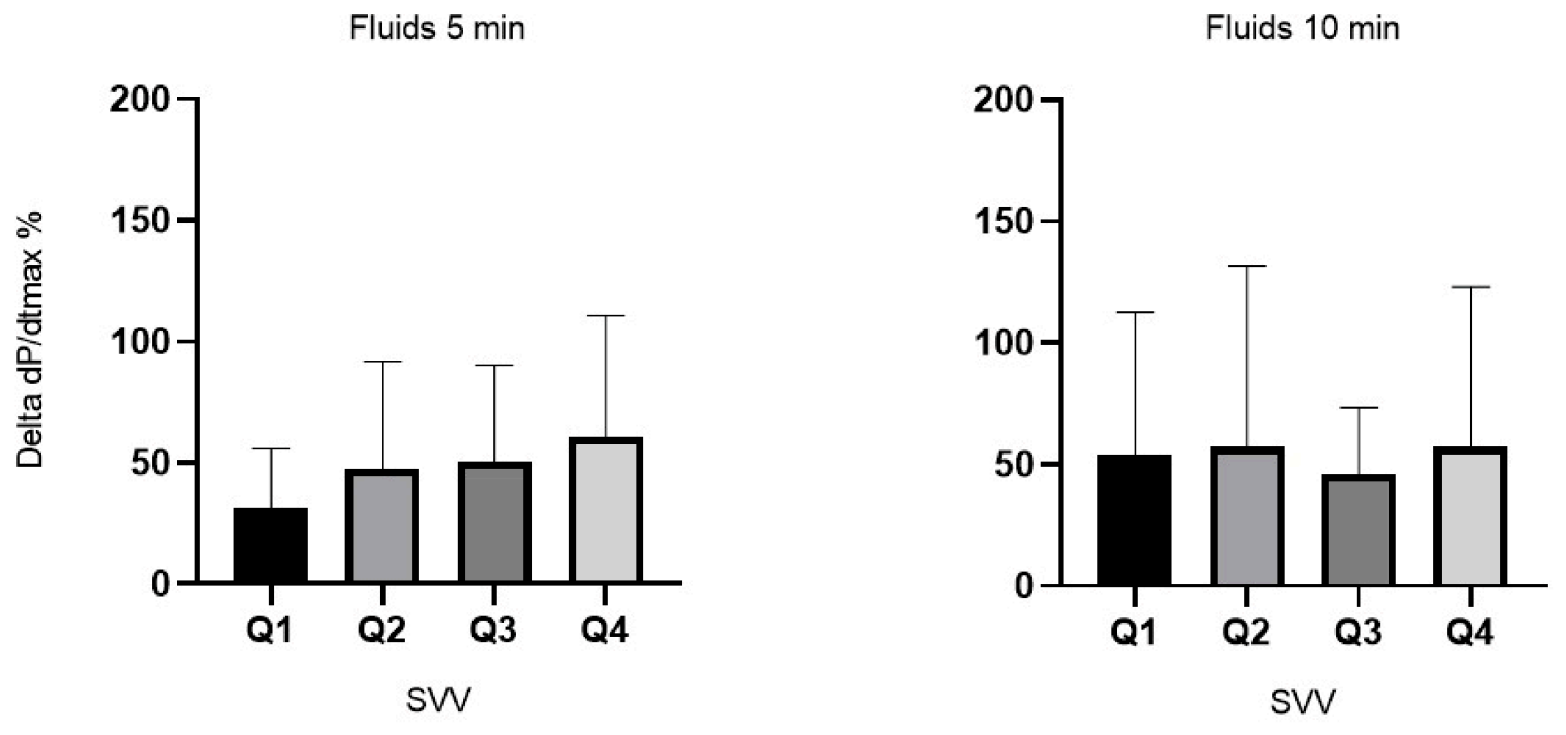
Observed within-episode percentage changes in arterial dP/dt_max_ at 5 and 10 min after fluid administration according to baseline stroke volume variation (SVV) quartiles. Box-and-whisker plots show the distributions of individual percentage changes across SVV quartiles (Q1–Q4). The figure is descriptive. Formal statistical inference was based on a linear mixed-effects model evaluating the baseline-to-5-minute change while accounting for repeated intervention episodes within patients.

In fluid-treated episodes, a linear mixed-effects model was used to evaluate arterial dP/dt_max_ across baseline SVV quartiles while accounting for repeated episodes within patients. Arterial dP/dt_max_ increased significantly from baseline to 5 min (time effect: F = 68.57, *p* < 0.001). However, neither the main effect of baseline SVV quartile (F = 0.09, *p* = 0.966) nor the SVV-quartile-by-time interaction (F = 0.51, *p* = 0.678) was significant. Thus, although arterial dP/dt_max_ increased after fluid administration, the magnitude of the early increase did not differ significantly among baseline SVV quartiles. The observed within-episode percentage changes at both 5 and 10 min are presented descriptively only in Figure 3.

### 3.2. Effects of Fluids on Haemodynamic Parameters

Following fluid administration, Stroke Volume (SV) increased progressively at 5 and 10 min, with median changes of 2.3% (*p* < 0.01) and 4.1% (*p* < 0.001), respectively [Figure S2]. The SVV decreased significantly (8.7% at 5 min and 18.8% at 10 min, both *p* < 0.001). SAP and DAP increased by 14% and 10.4% at 5 min and by 23% and 15.6% at 10 min, respectively (all *p* < 0.001). Heart rate (HR) showed a slight progressive increase (0% at 5 min, *p* = 0.36 and 1.1% at 10 min, *p* = 0.16). Ea_dyn_ remained unchanged at all timepoints (0% at 5 and 10 min, *p* = 0.17 and *p* = 0.08, respectively), while total arterial elastance (Eatot) increased by 10.3% at 5 min and 17.5% at 10 min (both *p* < 0.001) (Table 2).

### 3.3. Effects of NE on Haemodynamic Parameters

In NE-treated episodes, SV increased by 1.8% at 5 min (*p* = 0.05) and 3.1% at 10 min (*p* < 0.01). SVV remained unchanged at 5 min (0%, *p* = 0.9) and decreased at 10 min (−11%, *p* < 0.001). SAP significantly increased by 12% at 5 min and 20% at 10 min (both *p* < 0.001). Also, DAP significantly increased by 9.5% at 5 min and 16.5% at 10 min (both *p* < 0.001). HR showed small but significant variations (−1.8% at 5 min and −1.9% at 10 min, both *p* < 0.01). Ea_dyn_ remained stable at all timepoints (0% at 5 and 10 min, *p* = 0.56 and *p* = 0.16, respectively). Ea_tot_ increased by 9.1% at 5 min and 18% at 10 min (both *p* < 0.001) (Table 3).

## 4. Discussion

This study evaluated radial arterial dP/dt_max_ during fluid loading and norepinephrine administration as part of a structured haemodynamic optimisation protocol in major abdominal surgery. Under relatively stable preload conditions, radial arterial dP/dt_max_ may provide useful information regarding ventricular systolic performance, although its interpretation remains influenced by loading conditions and arterial properties. Intraoperative hypotension (IOH) is a frequent challenge in high-risk surgical patients, and advanced arterial waveform monitoring provides real-time insight into the components of circulatory failure. Differentiating whether hypotension is driven primarily by inadequate preload, impaired contractility, or reduced vascular tone is crucial to selecting the most appropriate therapeutic intervention. Among available strategies, fluid administration remains the most commonly applied treatment for IOH, as this is often related to relative hypovolemia caused by anaesthetic-induced vasodilation and fluid redistribution. Volatile anaesthetics, propofol, and opioids reduce sympathetic tone and venous return and may also exert direct negative inotropic effects [15,16,17,18,19]. This combination complicates the interpretation of intraoperative haemodynamic changes. Accurate assessment of ventricular systolic performance is therefore essential to avoid unnecessary fluid administration and to identify patients who may benefit from inotropic support. In this context, arterial dP/dt_max_ has been proposed as a real-time haemodynamic variable that reflects left ventricular systolic performance. However, its interpretation remains influenced by loading conditions, arterial properties, and ventriculo-arterial interaction and therefore should not be interpreted as a direct measure of intrinsic myocardial contractility. Ideally, an index of systolic performance should be sensitive to changes in myocardial contractility while minimising the influence of loading conditions [20]. García et al. demonstrated, in an experimental model under controlled loading and inotropic conditions, a close association between arterial dP/dt_max_ and end-systolic elastance (Ees), the reference load-independent index of systolic function [21]. These experimental findings support the physiological rationale for considering arterial dP/dt_max_ during haemodynamic assessment. However, extrapolation of these experimental observations to the intraoperative clinical setting requires caution, as preload, arterial load, vascular tone, and ventriculo-arterial coupling cannot be fully controlled and may substantially influence arterial dP/dt_max_. Because arterial dP/dt_max_ may be influenced by loading conditions, a low value (e.g., <400 mmHg·s^−1^) may reflect suboptimal ventricular filling rather than true myocardial contractile impairment, and a significant increase in dP/dt_max_ after fluid administration may merely reflect the Frank–Starling mechanism. Our findings reinforce the importance of assessing preload responsiveness before interpreting arterial dP/dt_max_ within the overall haemodynamic evaluation. When fluid-treated episodes were stratified according to baseline SVV quartiles, arterial dP/dt_max_ increased significantly after fluid administration. However, after accounting for repeated intervention episodes within patients, neither the baseline SVV quartile effect nor the SVV-quartile-by-time interaction was significant. Therefore, the magnitude of the early increase in arterial dP/dt_max_ did not differ significantly across baseline SVV quartiles. Taken together, these observations suggest that the early increase in arterial dP/dt_max_ following fluid loading is consistent with an acute preload-mediated response through the Frank-Starling mechanism. However, because preload, arterial load, and other determinants of arterial dP/dt_max_ cannot be completely separated in the present clinical setting, the contribution of additional physiological mechanisms cannot be excluded.

The mixed-effects model demonstrated that arterial dP/dt_max_ increased significantly after both protocol-guided interventions. Estimated mean increases were 114 mmHg·s^−1^ during fluid-treated episodes and 71.5 mmHg·s^−1^ during norepinephrine-treated episodes. The significant treatment-by-time interaction indicated that the temporal evolution of arterial dP/dt_max_ differed between the two algorithm-defined haemodynamic conditions while appropriately accounting for repeated intervention episodes within individual patients. The observed differences in arterial dP/dt_max_ between fluid-treated and norepinephrine-treated episodes should therefore be interpreted within the context of the predefined haemodynamic algorithm, which identified two distinct physiological conditions rather than comparable treatment groups. Accordingly, these findings describe physiological responses associated with different haemodynamic states and should not be interpreted as evidence of comparative treatment efficacy or superiority of one intervention over the other. Although the sensitivity analysis confirmed the robustness of the primary findings, it cannot fully account for residual confounding related to preload, arterial load, vascular tone, or other unmeasured physiological determinants.

In fluid-treated episodes, fluid administration produced marked reductions in SVV and increases in SV, consistent with effective preload recruitment. We hypothesise that the observed increase in arterial dP/dt_max_ may have been largely pressure-driven. However, the present study was not designed to formally evaluate the relative contributions of arterial pressure, stroke volume, heart rate, arterial elastance, or other determinants of arterial dP/dt_max_ to this response. Therefore, this physiological interpretation should be regarded as hypothesis-generating rather than as a demonstrated mechanism. Accordingly, increases in arterial dP/dt_max_ under preload-dependent conditions should not be interpreted as direct evidence of improved intrinsic myocardial contractility.

In contrast, during norepinephrine-treated hypotensive episodes triggered by low arterial pressure, with high SVV and low dynamic arterial elastance, arterial dP/dt_max_ showed a clear and progressive increase (approximately +13% at 5 min and +24% at 10 min). This increase occurred in the setting of marked rises in systolic and diastolic arterial pressure and total arterial elastance, while stroke volume changed only modestly. Previous experimental data suggest that arterial dP/dt_max_ becomes more interpretable as a marker of systolic performance when preload changes are limited [20]. Although the observed increase in arterial dP/dt_max_ occurred in parallel with marked changes in arterial pressure and arterial elastance, the present study cannot determine the relative contribution of arterial loading, ventricular performance, venous return, ventricular–arterial coupling, or direct β-adrenergic effects to this response [22,23]. Moreover, arterial load extends beyond arterial pressure alone and encompasses arterial compliance, vascular impedance, wave reflections, and ventriculo-arterial coupling, all of which may influence the morphology of the arterial pressure waveform and consequently arterial dP/dt_max_. These observations further support the interpretation of arterial dP/dt_max_ as an integrated haemodynamic variable rather than an isolated marker of myocardial contractility. In clinical practice, changes in arterial dP/dt_max_ are likely to arise from interactions among ventricular performance, loading conditions, arterial properties, and ventriculo-arterial coupling. This interpretation is consistent with recently proposed integrative haemodynamic frameworks, which emphasise that cardiovascular responses to interventions such as fluid administration or norepinephrine reflect the integrated interaction between multiple physiological interfaces rather than isolated changes in preload, afterload, or contractility [24]. Because these physiological interfaces were not directly assessed in the present study, their individual contribution to changes in arterial dP/dt_max_ cannot be determined. The end-systolic pressure–volume relationship (ESPVR) and its slope—end-systolic elastance (Ees)—remain the physiological gold standard for assessing ventricular contractility [25]. Nevertheless, contemporary perioperative haemodynamic optimisation protocols have not routinely incorporated arterial dP/dt_max_ into therapeutic decision-making, partly due to concerns about its load dependence. Only a few clinical studies have explored the integration of dP/dt_max_ into structured haemodynamic algorithms while explicitly accounting for preload status. Notably, Frassanito et al. proposed an HPI-guided protocol in which a dP/dt_max_ value <400 mmHg·s^−1^ prompted dobutamine administration exclusively in non-fluid-responsive conditions. However, the authors neither reported the distribution of dP/dt_max_ values nor evaluated its response to dobutamine, limiting direct comparison with other approaches [26]. To our knowledge, this is the first clinical study in major non-cardiac surgery to characterise arterial dP/dt_max_ responses to both fluid administration and NE boluses within an algorithm that explicitly accounts for preload dependency. Our findings support interpreting arterial dP/dt_max_ within an integrated haemodynamic assessment after preload dependence has been evaluated, rather than as a direct measure of intrinsic myocardial contractility. In fluid-treated hypotension, fluid boluses produced a consistent haemodynamic pattern, characterised by an increased stroke volume, decreased SVV, higher systolic arterial pressure, and a significant rise in arterial dP/dt compared to baseline. These changes align with an upward shift along the Frank–Starling curve, resulting in a larger systolic pressure waveform and a steeper systolic upstroke.

This study has several limitations. First, it was conducted in a single centre and included patients undergoing major open abdominal surgery, which may limit the generalisability of the findings to other surgical populations, minimally invasive procedures, or different haemodynamic contexts. Second, arterial dP/dt_max_ was derived from the radial artery waveform. Although previous studies have reported associations between radial arterial dP/dt_max_ and indices of ventricular systolic performance, the absolute values may differ across arterial sites. Moreover, arterial dP/dt_max_ is influenced by signal acquisition characteristics, including sampling frequency and waveform fidelity. The proprietary processing algorithms and sampling characteristics of the monitoring platform may therefore affect absolute dP/dt_max_ values and limit extrapolation to other devices. Third, the study design did not include a load-independent reference index of contractility (e.g., Ees), precluding direct validation of dP/dt_max_ against a gold standard. Nevertheless, preload dependency (SVV) and Ea_dyn_ were incorporated into the haemodynamic algorithm to mitigate the influence of loading conditions. Our findings support interpreting arterial dP/dt_max_ as an integrated haemodynamic variable once preload dependence has been evaluated and underscore the potential value of incorporating this parameter into structured haemodynamic optimisation strategies [27]. Furthermore, the study’s observational design does not allow the independent effects of preload, afterload, arterial impedance, ventricular–arterial coupling, and intrinsic myocardial contractility on arterial dP/dt_max_ to be separated. Consequently, changes in arterial dP/dt_max_ should be interpreted as reflecting the integrated cardiovascular response rather than isolated changes in myocardial contractility. In addition, right ventricular function and right ventricular–pulmonary vascular coupling were not assessed, and therefore their contribution to changes in arterial dP/dt_max_ cannot be evaluated. Finally, the study evaluated short-term haemodynamic responses (up to 10 min), and longer-term effects of repeated interventions were not assessed. Another limitation of the present study is the relatively positive intraoperative fluid balance, driven by substantial crystalloid administration despite limited blood loss and urine output. This was a direct consequence of the haemodynamic algorithm, which mandated fluid challenges whenever predefined indicators of preload dependency (SVV ≥ 13% and Ea_dyn_ ≥ 0.9) were met. Under general anaesthesia, factors such as vasodilation, surgical exposure, insensible losses, and fluid redistribution frequently met these criteria, leading to repeated fluid administration to maximise preload responsiveness. Although this approach resulted in a positive cumulative fluid balance, the volumes administered were consistent with historical practice and standard perioperative fluid management protocols at our institution. This strategy was associated with higher oxygen delivery and lower serum lactate concentrations at the end of surgery. However, these findings should be interpreted as physiological changes associated with protocol-guided haemodynamic management rather than as direct evidence of improved tissue perfusion, because the study did not include an untreated comparator and these variables may have been influenced by multiple perioperative factors. In conclusion, arterial dP/dt_max_ should be interpreted as an integrated haemodynamic variable influenced by ventricular performance, loading conditions, and arterial properties, rather than as a direct measure of intrinsic myocardial contractility. Future studies incorporating direct load-independent measurements of ventricular contractility are warranted to better define the physiological determinants of arterial dP/dt_max_ across different perioperative settings.

## Figures and Tables

**Table 1:** Baseline demographic variables and intraoperative management. Continuous variables (e.g., age, BMI, surgery duration, blood loss, urine output, administered fluid volumes, and length of hospital stay) are reported as median [IQR]. Categorical variables, including ASA physical status, blood transfusion, vasoactive drug administration, and postoperative destination, are presented as numbers (percentages), with administered doses reported only for recipients.

Variable	Value
Age (years)	70 [63–75]
BMI (kg/m^2^)	24 [22–27]
Male sex, n (%)	54 (61.4)
ASA physical status (I/II/III), n	0/49/39
Duration of surgery (min)	327 [271–418]
Blood loss (mL)	300 [200–500]
Urine output (mL)	600 [378–988]
Crystalloids (mL)	3500 [2500–4375]
Colloids, n (%)	42 (47.7)
Colloid volume in recipients (mL)	500 [250–750]
Blood transfusion, n (%)	17 (19.3)
Transfused volume in recipients (mL)	460 [250–500]
Ephedrine, n (%)	29 (32.9)
Ephedrine dose in recipients (mg)	10 [10–25]
Norepinephrine, n (%)	65 (73.9)
Norepinephrine dose in recipients (μg)	1000 [40–2400]
ICU, n (%)	22 (25.0)
PACU admission, n (%)	66 (75.0)
Length of hospital stay (days)	9 [8–11]

BMI—body mass index; ASA—American Society of Anesthesiologists; ICU—intensive care unit; PACU—post-anaesthesia care unit.

**Table 2:** Data are presented as median [interquartile range]. Percent changes (Δ%) represent the median of paired within-episode percentage changes calculated relative to the baseline value for each individual episode. Consequently, the reported Δ% values do not necessarily correspond to the percentage change calculated from the reported group medians. *p*-values refer to paired Wilcoxon signed-rank tests comparing each time point with the corresponding baseline measurement.

Variable	Baseline	5 min	Δ%	*p*-Value	10 min	Δ%	*p*-Value
**SV (mL)**	56.5 [49–66]	59 [51–69]	+2.3	<0.001	59 [51–72]	+4.1	<0.001
**SVV (%)**	15 [13.8–18]	14 [11–17]	−8.7	<0.001	13 [10–17]	−18.8	<0.001
**SAP (mmHg)**	84 [80–91]	99 [86–118]	+14.0	<0.001	104 [92–122]	+23.0	<0.001
**DAP (mmHg)**	49 [46–51]	54 [50–62]	+10.4	<0.001	56 [51–64]	+15.6	<0.001
**HR (bpm)**	75 [67–86]	76 [68–88]	0.0	0.36	76 [68–89]	+1.1	0.16
**Ea_dyn_**	1.2 [1.0–1.3]	1.2 [1.0–1.3]	0.0	0.17	1.2 [1.0–1.3]	0.0	0.08
**Eatot**	1.4 [1.1–1.5]	1.53 [1.3–1.8]	+10.3	<0.001	1.5 [1.4–1.9]	+17.5	<0.001

SV—stroke volume; SVV—stroke volume variation; SAP—systolic arterial pressure; DAP—diastolic arterial pressure; HR—heart rate; Ea_dyn_—dynamic arterial elastance; Eatot—total arterial elastance.

**Table 3:** Data are presented as median [interquartile range]. Percent changes (Δ%) represent the median of paired within-episode percentage changes calculated relative to the baseline value for each individual episode. Consequently, the reported Δ% values do not necessarily correspond to the percentage change calculated from the reported group medians. *p*-values refer to paired Wilcoxon signed-rank tests comparing each time point with the corresponding baseline measurement.

Variable	Baseline	5 min	Δ%	*p*-Value	10 min	Δ%	*p*-Value
**SV (mL)**	64 [54–75]	72 [63–81]	+1.8	<0.05	72 [63–83]	+3.1	<0.01
**SVV (%)**	13 [9–15]	9 [7–11]	0.0	0.90	8 [7–11]	−11.0	<0.001
**SAP (mmHg)**	88 [82–95]	101 [91–116]	+12.0	<0.001	109 [96–125]	+20.0	<0.001
**DAP (mmHg)**	47 [44–49]	51 [46–57]	+9.5	<0.001	55 [48–61]	+16.5	<0.001
**HR (bpm)**	69 [59–79]	66 [58–78]	−1.8	<0.01	66 [58–74]	−1.9	<0.01
**Ea_dyn_**	1.0 [0.8–1.2]	1.0 [0.9–1.2]	0.0	0.56	1.0 [0.8–1.2]	0.0	0.16
**Eatot**	1.2 [1.0–1.3]	1.3 [1.1–1.5]	+9.1	<0.001	1.4 [1.2–1.6]	+18.0	<0.001

SV—stroke volume; SVV—stroke volume variation; SAP—systolic arterial pressure; DAP—diastolic arterial pressure; HR—heart rate; Ea_dyn_—dynamic arterial elastance; Eatot—total arterial elastance.

## Data Availability

The data presented in this study are available upon request from the corresponding author. The data are not publicly available due to privacy and ethical restrictions.

## Supplementary Materials

The following supporting information can be downloaded at https://www.mdpi.com/article/10.3390/jcm15166471/s1, Figure S1: Scatter plot showing individual arterial dP/dt_max_ values at baseline (0), 5 min, and 10 min after ephedrine administration in hypotensive, non-fluid-treated episodes (MAP < 65 mmHg, SVV < 13%, dP/dt_max_ < 400 mmHg·s^−1^ at baseline). Horizontal bars represent mean values; Figure S2: Relationship between percentage changes in stroke volume (ΔSV%) and percentage changes in dP/dt (ΔdP/dt%) following protocol-guided fluid administration.
