# Supplementary material for: Arterial dP/dtmax Response to Fluids and Norepinephrine During Intraoperative Hypotension: A Prospective Physiological Study
Source: J Clin Med. 2026 Aug 21;15(16):6471. doi: 10.3390/jcm15166471 (PMC13514141; doi:10.3390/jcm15166471)
Supplement: Supplementary file 1 [file jcm-15-06471-s001.zip › jcm-4422096-supplementary.pdf]

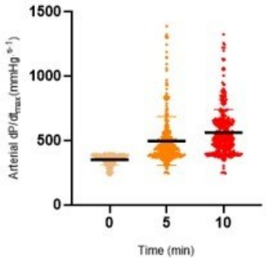

Figure S1. Scatter plot showing individual arterial dP/dtmax values at baseline (0), 5 minutes, and 10 minutes after ephedrine administration in hypotensive, non–fluid-responsive episodes (MAP <65 mmHg, SVV <13%, dP/dtmax <400 mmHg·s^{-1} at baseline). Horizontal bars represent mean values.

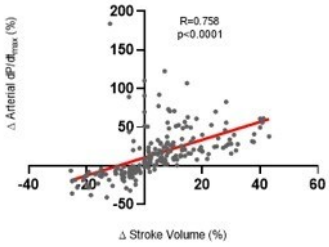

Figure S2. Relationship between percentage changes in stroke volume (ΔSV%) and percentage changes in dP/dt (ΔdP/dt%) following protocol-guided fluid administration.
